# Persistent Allergic Rhinitis and the XPERT Study

**DOI:** 10.1097/1939-4551-4-S3-S32

**Published:** 2011-03-15

**Authors:** Anthi Rogkakou, Elisa Villa, Valentina Garelli, G Walter Canonica

**Affiliations:** 1 Allergy and Respiratory Diseases, Department of Internal Medicine, University of Genoa, Genoa, Italy

**Keywords:** persistent allergic rhinitis, XPERT Study, ARIA, PER, treatment

## Abstract

Allergic rhinitis (AR) is a chronic disease with an increasing trend in most of the Western Countries. It may significantly impair the individual quality of life (QoL) and also represents a social burden for its economic costs. Levocetirizine (XYZAL; UCB Pharma) as a second generation, nonsedating H1-antihistamine, has been shown to be clinically effective in patients with AR in different randomized controlled trials. The XPERT (XYZAL in Persistent Rhinitis Trial) is the first large, long-term clinical study involving patients with persistent rhinitis as defined by ARIA (Allergic Rhinitis and its Impact on Asthma). The XPERT was a 6-month double-blind, placebo-controlled, multicenter, multinational trial in 551 subjects. Adults with persistent rhinitis sensitized to both grass pollen and house dust mites were randomized to receive levocetirizine 5 mg/d or placebo. Two primary objectives were considered: comparison of the Rhinoconjunctivitis Quality of Life Questionnaire (RQLQ) overall score and Total 5 Symptoms Score (rhinorrhea, sneezing, nasal congestion, and nasal and ocular pruritus) (T5SS) between active and control group over a period of 4 weeks. As secondary endpoints, similar evaluations at 1 week and 3, 4, 5, and 6 months, summary scores for a general health status questionnaire (Medical Outcomes Survey Short Form 36), comorbidities, pharmacoeconomic and safety evaluations. Levocetirizine significantly improved both the RQLQ overall score and the T5SS from week 1 to 6 months (*P *< .001). Medical Outcomes Survey Short Form 36 summary scores were also improved in the group treated with levocetirizine with respect to placebo. Treatment cessation because of lack of efficacy, comorbidities, and overall costs of disease, and comorbidities per working patient per month (160.27 vs 108.18) were lower in the levocetirizine group. In conclusion, levocetirizine resulted to improve the quality of life and the symptoms related to AR and also to reduce the overall costs of the disease after 6 months treatment.

## 

Allergic rhinitis is a frequent chronic disease that may significantly impair health-related quality of life (HRQoL). Several studies and epidemiological data have shown that allergic rhinitis has an economically remarkable impact caused by work days loss, absenteeism, and poor task performance, with a global reduction in productivity. It is fundamental to remember the ARIA document published in 2001[1] where allergic rhinitis is divided into 4 categories: mild intermittent, moderate/severe intermittent, mild persistent, and moderate/severe persistent (depending on severity and duration of symptoms and quality of life). This classification has been recently confirmed at an international level[2] and a stepwise pharmacologic treatment is proposed on the basis of ARIA criteria. In all the categories mentioned before, a treatment based on the use of a second-generation nonsedating H_1_-antihistamine is recommended for the management of allergic rhinitis, according to ARIA guidelines.

Levocetirizine (XYZAL; UCB Pharma, Brussels, Belgium) is an oral, nonsedating H_1_-antihistamine that proved to be significantly effective in improving symptoms in patients with allergic rhinitis; it presents a good safety profile[3-8] and, for all these pharmacologic characteristics, is highly indicated as a first-line treatment in subjects with persistent allergic rhinitis (PER). The XYZAL in Persistent Rhinitis Trial (XPERT) is a remarkable study published in 2004,[9] providing different clinical, HRQoL, and pharmacoeconomic assessments, applied over the long term, and using electronic diary cards, to evaluate the efficacy of levocetirizine in both HRQoL and economic aspects.

Two primary outcomes were considered in the trial:

• To analyze and compare the effects of levocetirizine versus placebo on HRQoL, as evaluated through the Rhinoconjunctivitis Quality of Life Questionnaire (RQLQ) overall score at baseline and after 4 weeks of treatment;

• To compare the mean Total 5 Symptoms Score (T5SS; sum of rhinorrhea, sneezing, nasal congestion, and nasal and ocular pruritus; score, 0-15), assessed for 24 hours over a period of 4 weeks of treatment.

Secondary objectives of the study were to compare RQLQ overall score and symptom scores after 1 week and 3, 4.5, and 6 months of treatment. Other secondary objectives were as follows:

• The comparison of the effects on health status as measured by the Medical Outcomes Survey Short Form 36 (SF-36) questionnaire (physical and mental summary scores) after periods of 4 weeks, 3 months, 4.5 months, and 6 months;

• The evaluation of the rescue medication use over the 6-month treatment period;

• The assessment of levocetirizine safety during the 6-month trial period.

The present survey also provided a relevant analysis of pharmacoeconomic data about the treatment of PER. It is a large multicenter, randomized, placebo-controlled, double-blind, parallel group trial, involving more than 500 patients from 63 centers in 5 European countries (Belgium, France, Germany, Italy, and Spain); in addition, it is the first long-term (6-month) trial carried out with an oral H_1_-antihistamine in PER, with important consequential implications on guide-lines criteria use and clinical practice.

In the study, all the patients enrolled were 18 years old at least and presented symptoms related to PER (defined as allergic rhinitis symptoms lasting 4 days or more per week for 4 consecutive weeks or more per year). They also presented positive skin prick test or specific serum IgE (CAP System; Pharmacia Diagnostic, Uppsala, Sweden) for house dust mites and one pollen allergen (grass or *Parietaria*, IgE level >3.5 U/mL) at least. Informed consent was obtained from all participants and the study was conducted according to good clinical practice and the Helsinki Declaration in 1996 and under permission of the respective institutional review boards. Demographic characteristics of the patients are described in Table 1.

**Table 1:** Demographic Characteristics of the Population

	**Placebo (*N ***= **273)**	**Levocetirizine (5 mg) (*N ***= **278)**	Total* (*N *= **551)**
Age at randomization, years			
Mean (SD)	30.8 (8.8)	29.8 (8.9)	30.3 (8.9)
Median	29.0	28.0	28.2
Minimum-maximum	18.1-70.3	18.0-66.2	18.0-70.3
Duration of PER before randomization, years, mean (SD)	12.8 (8.2)	11.9 (7.8)	12.3 (8.0)
Sex, female	158 (57.9%)	152 (54.7%)	310 (56.3%)
Working status			
Working	196 (72%)	186 (67%)	382 (69%)
Nonworking	77 (28%)	92 (33%)	169 (31%)

^*^ITT population.

Globally considered, 76.4% of patients completed the study treatment, 80.9% levocetirizine and 71.8% placebo. Forty-five patients (16.5%) in the placebo group dropped out for absent or insufficient efficacy, with respect to 21 (7.6%) in the levocetirizine group (*P *= 0.0007 according to the Wilcoxon test). Other reasons for dropout were represented by withdrawal of consent for personal reasons (15, 5.5% placebo patients; 17, 6.1% levocetirizine patients), adverse events (8, 2.9% placebo; 11, 4.0% levocetirizine), and others (8, 2.9% placebo; 4, 1.4% levocetirizine). One patient receiving placebo was lost to follow-up.

Patients were enrolled at a randomization visit, after 1 week, if they presented a T5SS >6 of 15 for at least 4 days during the run-in period. All the randomized patients received 5 mg of levocetirizine or placebo orally each day, starting on the evening of the second visit and continuing for 6 months. All the patients included in the study were evaluated at the end of the first treatment week and again at weeks 4, 12, 18, 26, and 27, after another week's follow-up.

Patients were excluded if they were pregnant or nursing mothers, women not using a medically accepted method of contraception, patients with ear, nose, or throat or eye infection during the 2 weeks preceding the first visit, and patients with asthma treated daily with other than an inhaled *β*-agonist on demand. Patients that presented atopic dermatitis or urticaria requiring antihistamine or corticosteroid treatment, with other ear, nose, or throat diseases such as vasomotor rhinitis or nasal polyps, other clinically significant diseases such as glaucoma or cardiovascular or hepatic diseases, or any disorder disturbing absorption, distribution, metabolism, or excretion of levocetirizine, were also excluded.

All the patients could use nasal or ocular cromoglycate and, in case of unbearable impairment of the allergic rhinitis symptoms, after a minimum of 4 weeks of treatment, were permitted a maximum of 20 mg of oral prednisolone once daily for 5 days, for a maximum of 2 prednisolone courses during the whole study. Compliance for both study and rescue medications was evaluated at each visit.

Health-related quality of life is an important parameter because it considers the effect of both disease and treatment on a patient's life as subjectively perceived.

The RQLQ is a disease-specific, validated, and reproducible tool for assessing HRQoL and how symptoms and treatments affect patients' physical, social, and emotional status[10]. It is characterized by 28 items and 7 domains. All of the items have a score ranging from 0 (no trouble) to 6 (major impairment). The RQLQ was completed at visit 2 (randomization) and at visits 3 to 7, or at the end of the study treatment in case of withdrawal.

The SF-36 is another well-validated questionnaire to evaluate health status, to assess the impact of various diseases, such as allergic rhinitis,[11] and to compare the effects of various treatments. Through 36 questions, it measures 8 health dimensions domains condensed into 2 physical and mental health summary measures, with scores ranging from 0 (worst reported health status) to 100 (best reported health status)[12]. All the patients had to complete the SF-36 at each visit, except visit 3 (week 1), after the RQLQ.

Rhinitis symptoms were assessed by using the T5SS. This total symptom score measures the symptoms normally responding to antihistamines, such as rhinorrhea, sneezing, nasal and ocular pruritus, and also nasal congestion, which is one of the most bothersome symptoms and maybe the most clinically relevant symptom in patients with PER. Through the use of electronic diaries, all the patients recorded their rhinitis symptoms daily, with 4-point scale score ranging from 0 (absent) to 3 (severe) for each symptom (Minidoc; Arracel, Sittingbourne, United Kingdom).

The authors also considered the pharmacoeconomic aspect, including estimates for direct medical cost and indirect cost in the working population due to PER and to comorbidities such as asthma, sinusitis, otitis media, and upper respiratory tract infections. Direct costs consisted of the use of medical resources: hospitalization, physician visits, and concomitant medications. Indirect costs associated with PER or comorbidities, assessed through a weekly questionnaire on Minidoc, included absenteeism and presenteeism (that is, lost of productivity while still present at work). UCB Pharma clinical trials operations monitored the whole study and helped in providing the statistical analyses.

A total of 724 patients were included at the screening visit, of whom 551 patients were randomized to received levocetirizine or placebo treatment.

Regarding T5SS, a statistically significant difference was observed for nasal and ocular symptoms just from the first week after starting the treatment; they remained stable for the entire duration of the 6-month treatment period. Nasal congestion was improved significantly after the first month of treatment and continued for >6 months (Figure 1)[13]. 

**Figure 1:**
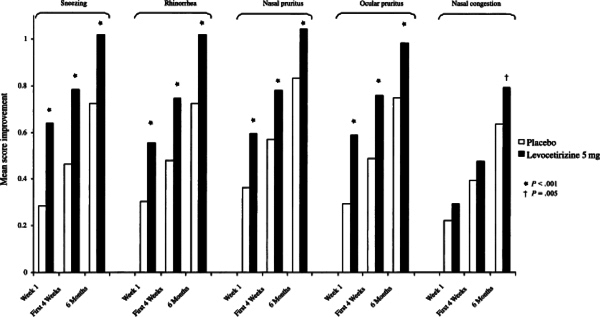
**Change in individual symptom scores over a period of 6 months**.

The use of cromoglycates, nasal and/or ocular, as rescue medications in case of allergic rhinitis impairment, was higher in the placebo group during the first 4 weeks (62.6% of the patients receiving placebo versus 49.3% of the patients treated with levocetirizine; *P *= 0.002); moreover, an increasing trend of rescue medication use was observed in the placebo group over the entire 6 months of treatment: 13.6% versus 10.8% for prednisolone (*P *= 0.362) and 75.8% versus 69.4% (*P *= 0.104) for cromoglycates.

Levocetirizine improved QoL assessed by the RQLQ questionnaire and SF-36 as early as the first week of treatment. This improvement of the levocetirizine group versus placebo was confirmed for each RQLQ domain (Table 2, Table 3). SF-36 summary scores followed a similar course[9]. In the group treated with levocetirizine, mean changes from baseline of physical and mental summary scores ranged from 3.65 to 5 and from 3.83 to 5.97, respectively, with respect to the placebo group where they ranged from 1.61 to 3.37 and from 2.79 to 3.99. Between the two study groups, significant differences were recorded about mean changes after 3 and 4.5 months for the mental component summary score (both *P *values <0.01) and after 4 weeks and 3, 4.5, and 6 months for the physical component summary score (all *P *values <0.01). In a parallel manner to the RQLQ overall score, subjective improvement in the health condition seemed related to symptoms relief.

**Table 2:** T5SS and RQLQ Overall Score at 4 Weeks

Treatment Group	N*	Baseline/Mean (SD)	After 4 Weeks of Treatment/Adjusted Mean (SEM)†	Difference vs Placebo (95% CI)/Adjusted Mean	*P*
RQLQ overall score					
Placebo	252	3.06 (0.94)	−1.01 (0.07)		
Levocetirizine (5 mg)	257	3.04 (0.92)	−1.49 (0.07)	0.48 (0.29-0.67)	<0.001
T5SS					
Placebo	271	8.90 (2.26)	−2.40 (0.15)		
Levocetirizine (5 mg)	276	9.02 (2.28)	−3.54 (0.15)	1.14 (0.75-1.52)	<0.001

^*^Number of ITT patients with nonmissing values at baseline and under treatment.

^†^Mean change from baseline, adjusted for baseline score and country.

**Table 3:** Changes of the RQLQ Domains at 4 Weeks From Baseline

	Placebo Change		Levocetirizine (5 mg) Change		Difference versus Placebo (95% CI)	
			
Domain	N*	Adjusted Mean† (SEM)	N*	Adjusted Mean† (SEM)	Adjusted Mean	*P*
Activities	241	−1.36 (0.10)	248	−2.08 (0.10)	0.73 (0.47-0.99)	<0.001
Emotions	252	−0.81 (0.07)	257	−1.16 (0.07)	0.35 (0.17-0.54)	<0.001
Eye symptoms	252	−0.91 (0.09)	257	−1.40 (0.09)	0.48 (0.26-0.70)	<0.001
Non-hay fever symptoms	252	−0.83 (0.08)	257	−1.21 (0.08)	0.38 (0.18-0.57)	<0.001
Nasal symptoms	252	−1.10 (0.09)	257	−1.64 (0.09)	0.54 (0.31-0.77)	<0.001
Practical problems	252	−1.50 (0.10)	257	−2.06 (0.10)	0.56 (0.30-0.82)	<0.001
Sleep	252	−0.86 (0.09)	257	−1.35 (0.09)	0.50 (0.27-0.73)	<0.001

*Number of ITT patients with nonmissing values at baseline and under treatment.

†Mean change from baseline, adjusted for baseline score and country.

Comorbidities were considered during the study: the most frequent were represented by upper respiratory infections and asthma. In an exploratory analysis of the XPERT study, levocetirizine reduced the percentage of patients with at least one asthma event to 7.4 (from 13.6 for placebo, *P *= 0.04) and reduced the mean number of asthma medication events from 0.23 per placebo patient to 0.11 (*P *< 0.001)[13].

Levocetirizine was well tolerated and safe for a greater than 6-month period, making it suitable for chronic treatment. In the placebo group, 193 (70.7%) patients, with respect to 192 (69.1%) of the active group, recorded at least one adverse event at some point during the 6-month study. Adverse events were commonly represented by headache (23.2% placebo versus 24.5% levocetirizine), pharyngitis (20.5% versus 19.8%), influenza-like symptoms (13.9% versus 14.0%), fatigue (7.0% versus 8.6%), sleepiness (1.8% versus 6.8%) and gastroenteritis (5.1% versus 2.9%). No hospitalizations occurred.

Levocetirizine treatment also caused a reduction of the overall cost of the disease. All the cost parameters (direct and indirect) in fact presented a significant improvement in patients treated with levocetirizine versus placebo group. A long-term levocetirizine therapy presented lower costs compared with those of the untreated PER and its comorbidities (€350 per patient per month). Loss of work days was lower in levocetirizine than placebo group (0.18 days per patient per month versus 0.45 days per patient per month). Levocetirizine treatment produced cost savings to society of more than €150 per patient per month, consequent to patients' major ability to maintain work and daily activities (Table 4)[14].

**Table 4:** Mean Direct and Indirect Costs Per Month Per Working Patient

	**Placebo Mean € (95% CI) (*N ***= **196)**	**Levocetirizine (5 mg) Mean € (95% CI) (*N ***= **186)**	Difference versus Placebo	*P*
Direct costs				
Total direct medical costs for PER	5.32€ (4.43, 6.42)	16.81€ (15.94, 18.13)	11.50€ (10.06, 13.03)	<0.001
Total direct medical costs for comorbidities	2.72€ (1.82, 4.20)	1.77€ (1.14, 3.04)	−0.96€ (−2.60, 0.40)	0.18
Indirect costs				
Absenteeism	45.70€ (32.02, 75.00)	18.57€ (13.75, 26.00)	−27.14€ (−55.78, −12.10)	<0.001
Presenteeism	106.54€ (86.97, 132.86)	71.04€ (56.16, 92.43)	−35.50€ (−65.21, −7.01)	0.02
Total costs	160.27€ (129.93, 204.54)	108.18€ (91.55, 131.78)	−52.09€ (−98.18, −13)	

In conclusion, XPERT was the first prospective study of levocetirizine in ARIA-defined PER. This multicenter, multinational, randomized, double-blind, placebo-controlled, parallel-group study was innovative not only in terms of definition of allergic rhinitis but also for the large sample (550 patients), long-lasting treatment period (26 weeks), the study design, and the aim of the study (clinical efficacy, QoL, adverse events, comorbidities, and pharmacoeconomy/economic costs). Furthermore, it was closer to the real life and PER study model with respect to previous trials. PER frequently has debilitating symptoms that interfere with patients QoL, their daily activities, and their productivity. A rapid onset of action, effective and long-lasting treatment like levocetirizine appears to have, is very important for a satisfying treatment of PER. Levocetirizine is effective against all the symptoms related to allergic rhinitis and seems also able to reduce the minimal persistent inflammation characterizing patients' mucosal structures affected by PER.

Levocetirizine is well-tolerated, safe, and suitable for continuous and long-lasting treatment. Furthermore, a long-term treatment with levocetirizine reduces overall costs (direct and indirect costs) for both PER and associated comorbidities, with a consequently important impact on socioeconomic aspects.

Levocetirizine thus satisfies all the ARIA/EAACI efficacy and safety criteria and is suitable for long-term treatment of PER as a first-line drug among the second-generation antihistamines.
